# Sleep Duration/Quality With Health Outcomes: An Umbrella Review of Meta-Analyses of Prospective Studies

**DOI:** 10.3389/fmed.2021.813943

**Published:** 2022-01-20

**Authors:** Chang Gao, Jiao Guo, Ting-Ting Gong, Jia-Le Lv, Xin-Yu Li, Fang-Hua Liu, Meng Zhang, Yi-Tong Shan, Yu-Hong Zhao, Qi-Jun Wu

**Affiliations:** ^1^Clinical Research Center, Shengjing Hospital of China Medical University, Shenyang, China; ^2^Department of Clinical Epidemiology, Shengjing Hospital of China Medical University, Shenyang, China; ^3^Department of Oncology, Shengjing Hospital of China Medical University, Shenyang, China; ^4^Department of Obstetrics and Gynecology, Shengjing Hospital of China Medical University, Shenyang, China; ^5^Department of Statistics, University of Washington, Seattle, WA, United States

**Keywords:** health outcomes, meta-analysis, sleep duration, sleep quality, umbrella review

## Abstract

**Background:**

To quantitatively evaluate the evidence of duration and quality of sleep as measured by multiple health outcomes.

**Methods:**

This review is registered with PROSPERO, number CRD42021235587. We systematically searched three databases from inception until November 15, 2020. For each meta-analysis, the summary effect size using fixed and random effects models, the 95% confidence interval, and the 95% prediction interval were assessed; heterogeneity, evidence of small-study effects, and excess significance bias were also estimated. According to the above metrics, we evaluated the credibility of each association.

**Results:**

A total of 85 meta-analyses with 36 health outcomes were included in the study. We observed highly suggestive evidence for an association between long sleep and an increased risk of all-cause mortality. Moreover, suggestive evidence supported the associations between long sleep and 5 increased risk of health outcomes (stroke, dyslipidaemia, mortality of coronary heart disease, stroke mortality, and the development or death of stroke); short sleep and increased risk of overweight and/or obesity; poor sleep quality and increased risk of diabetes mellitus and gestational diabetes mellitus.

**Conclusions:**

Only the evidence of the association of long sleep with an increased risk of all-cause mortality was graded as highly suggestive. Additional studies are needed to be conducted.

**Systematic Review Registration:** https://www.crd.york.ac.uk/PROSPERO/, identifier: CRD42021235587

## Introduction

Sleep is an important and complex physiological process for maintaining optimal health. The National Sleep Foundation recommends 7–9 h of sleep for people aged 26–64 years and 7–8 h of sleep for people aged ≥65 years (1). However, because of irregular working, shift-work patterns, and unhealthy sleeping habits, the quantity and quality of sleep may be abnormal in modern society. Over the last few decades, there has been growing evidence to suggest that self-reported short or long sleep duration (often defined as <6 or 7 and >8 or 9 h, respectively) and poor sleep quality [Pittsburgh Sleep Quality Index (PSQI) > 5] may be consistently associated with adverse health outcomes [e.g., short sleep and increased risk of hypertension (2), long sleep and increased risk of chronic kidney disease (3), poor sleep quality and increased risk of preterm birth (4), etc.].

Although previous studies have examined this topic using various methodologies, a quantitative appraisal of epidemiological credibility is lacking, as are examinations of the potential bias between the quantity and quality of sleep and health-related outcomes and assessments of the most influential outcomes. Therefore, in the present study, we conducted an umbrella review of the evidence between the quantity and quality of sleep and the multiple health outcomes in systematic reviews and meta-analyses, assessed the diverse bias, and quantitatively evaluated the strength and credibility of the evidence.

## Methods

We strictly followed standardized guidelines to perform an umbrella review, which is the systematic collection and evaluation of multiple systematic reviews and meta-analyses conducted on a specific research topic (5). The umbrella review was conducted according to the Preferred Reporting Items for Systematic Reviews, Meta-Analyses guidelines (Supplementary Table S1) (6), and the guidance of the Meta-analysis of Observational Studies in Epidemiology statement (Supplementary Table S2) (7). The study protocol was registered in the PROSPERO database for systematic reviews and meta-analyses (registration number: CRD42021235587).

### Search Strategy

The electronic databases, PubMed, EMBASE, and the Web of Science were searched systematically from inception until November 15, 2020, to identify related systematic reviews and meta-analyses of observational studies. A predefined search strategy was used, which is presented in Supplementary Table S3. In addition, we performed a manual check of reference lists from the retrieved articles for further potentially relevant articles.

### Eligibility Criteria and Appraisal of Included Studies

Two authors (CG and X-YL) independently scrutinized articles based on titles and abstracts. If needed, full articles were retrieved for a final decision. Disagreements between the two reviewers were resolved by discussion and consensus with a senior advisor (T-TG). Articles were included according to the following criteria: (A) systematic reviews and meta-analyses of prospective studies on the associations between duration and quality of sleep and any health-related outcome, (B) studies that relied on data from human studies with any type of health-related outcome measure, and (C) studies that reported effect sizes such as odds ratios (ORs), relative risk (RRs), or hazard ratios (HRs) at follow-up. We included information that we were interested in in each study, such as subgroup analysis and dose–response analysis. If a systematic review or meta-analysis performed a subgroup analysis stratified by the study design, then the results for prospective studies were included (8–10).

We excluded individual studies according to the following criteria: (A) meta-analyses of case-control or cross-sectional studies, (B) studies in which sleep measures were not the exposure of interest (such as sleep-disordered breathing, restless leg syndrome, or napping), (C) meta-analyses or systematic reviews that did not present study-specific data [effect sizes, 95% confidence intervals (CIs) and numbers of cases/population)], (D) systematic reviews without a quantitative synthesis, or (E) other types of papers (e.g., review, abstract, non-English, or editorial). For the main analysis, whenever an eligible meta-analysis included a lower number of component studies compared to other meta-analyses related to the same association, we retained the one with the largest number of primary studies (8–10).

### Exposure Identification

For exposures, the studies of sleep duration included “short sleep duration” and “long sleep duration.” In most studies, “sleep duration” was defined as hours per day or minutes per night. “Short sleep duration” was defined as ≤ 5, <5, ≤ 6, <6, ≤ 5–6 or <7, and “long sleep duration” was defined as >7, ≥8, ≥9 or ≥8–9 h. The reference categories for sleep duration in the studies, in h per night, were 7, 7–8, 6–8 or 7–9 h. In this umbrella review, “poor sleep quality,” “good sleep quality” and “not-poor sleep quality” were separately characterized as PSQI > 5, PSQI <5 and PSQI ≤ 5.

### Data Extraction

Two investigators (X-YL and F-HL) independently extracted the related data from the included studies using a custom-made data extraction form. In the case of discrepancies, the data were subsequently verified by a third author (CG). The data-collection form included the first author, year of publication, journal of publication, exposure, outcome examined, number of included studies, case number, and study population. For each of the included studies in each eligible meta-analysis, we extracted the first author, year of publication, epidemiological design, number of cases and total population, and the maximally adjusted relative risk (ORs, RRs or HRs) along with the corresponding 95% CI.

### Data Analysis

For each exposure and outcome pair, we evaluated the summary effect size and the 95% CI through both fixed and random effects models (11, 12). The heterogeneity between studies was assessed with the I^2^ metric of inconsistency and its 95% CI (13). The I^2^ ranges between 0 and 100% and quantifies the variability in effect estimates that it is due to heterogeneity rather than sampling error. Values exceeding 50% were indicative of high heterogeneity, whereas values >75% implied very high heterogeneity (14). We also calculated the 95% prediction interval (PI), which further accounted for heterogeneity between studies and estimated the uncertainty of the association if future studies examine that same association (15).

We used Egger's regression asymmetry test to identify small-study effects (16) to evaluate whether smaller studies tend to give higher risk estimates compared with larger studies, which can indicate publication, other reporting biases, or other reasons for differences between small and large studies (17). We calculated the standard error of the effect size for the largest data set of each meta-analysis to determine whether larger estimates of effect size were predicted by small studies compared to large studies (10). Indication of small study effects was based on the *P* value for Egger's test was smaller than 0.10 and the largest study had a smaller effect size than the summary effect size (17).

We applied the excess significance test to evaluate whether the observed number of studies (O) with statistically significant results among those included in a meta-analysis was larger than the expected number of studies (E) with statistically significant results (18). E is calculated by the sum of the statistical power estimates for each component study. The statistical power of each study was calculated with an algorithm using a non-central *t* distribution (19). The excess significance test for single meta-analyses was considered positive at *P* < 0.10, given that O > E as previously proposed (10). When standardized mean differences were reported, we planned to transform these estimates into ORs (20). The statistical analysis and the power calculations were conducted in STATA version 15.0, and all *P* values were two-tailed.

### Methodological Quality Appraisal

To study the quality of the reporting of the included systematic reviews and meta-analyses, two investigators (CG and X-YL) independently rated the methodological quality with the Assessment of Multiple Systematic Reviews (AMSTAR-1) tool. Higher scores imply greater quality, ranging from 0 to 11. The AMSTAR-1 tool involves dichotomous scoring (0 or 1) of 11 related items to assess the methodological rigor of the included articles, such as a comprehensive search strategy or publication bias assessment. AMSTAR-1 scores are graded as high (8–11), medium (4–7), and low quality (0–3) (21).

### Grading the Evidence

Statistically significant meta-analyses (*P* < 0.05) were rated into four levels (convincing, highly suggestive, suggestive, and weak) using specific criteria. For convincing evidence: *P* < 10^−6^, number of cases >1,000, I^2^ < 50%, *P* < 0.05 of the largest component study in the meta-analysis, 95% PI excludes the null value, absence of small-study effects (*P* > 0.1 for Egger's test), and no excess significance bias (*P* > 0.1). For highly suggestive evidence: *P* < 10^−6^, number of cases >1,000, and *P* < 0.05 of the largest study. For suggestive evidence: *P* < 10^−3^, and number of cases >1,000. For weak evidence, the sole criterion was *P* < 0.05 (22). When *P* > 0.05, there was no association (10). All analyses were conducted in STATA, version 15.0.

## Results

### Study Selection

**Figure 1:**
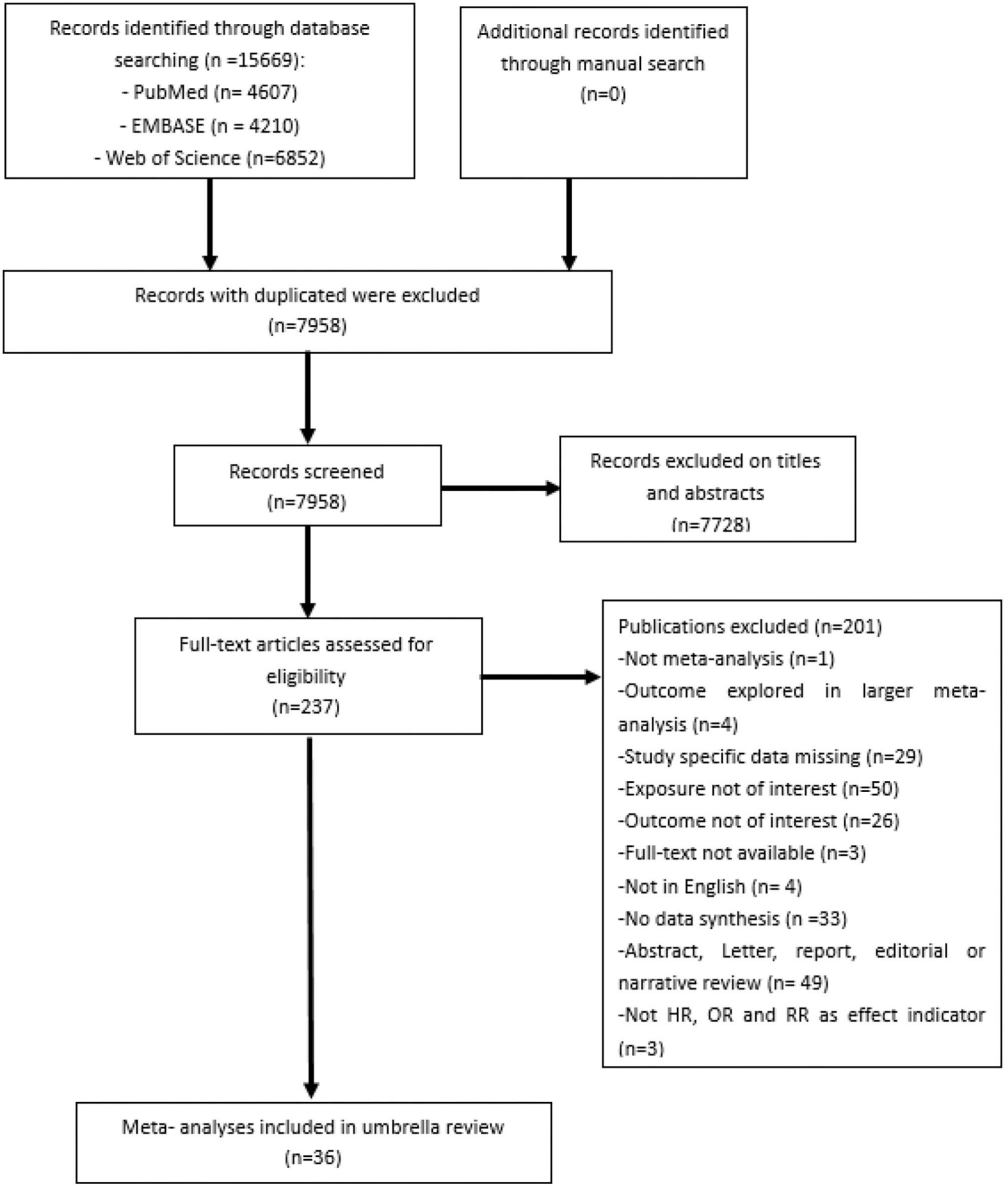
Flow diagram of the study selection process.

As reported in Figure 1, 15,669 records were retrieved across three electronic databases search, and 7,958 records were identified unduplicated through the parallel reviews. A total of 7,728 records were excluded after title and abstract screening, and 201 were excluded through assessment of the full-text (Figure 1). Ultimately, 36 articles were included in our umbrella review for analysis.

### Characteristics of Included Meta-Analyses

The characteristics of these 36 articles are summarized in Table 1. All articles were published between 2009 and 2020. These included studies covered 85 meta-analyses, which reported associations between duration and quality of sleep and 36 different outcomes. The median number of original studies in each meta-analysis was 7 (range from 3 to 27), while the median number of cases was 4,848 (range from 156 to 219,518), and the median number of the total participants was 113,226 (range from 1,230 to 2,311,390). The case number exceeded 1,000 in 81 meta-analyses.

**Table 1:** Main characteristics of included systematic reviews or meta-analyses that evaluate sleep duration/quality and health outcome risk.

**Outcomes**	**Individual study**	**No. of studies**	**Effect metric**	**Level of comparison**	**Summary effect size (95% CI)**	
					**Random effects**	**Fixed effects**
**Sleep duration**						
**Diseases of the circulatory system**						
Atrial fibrillation	Chokesuwattanaskul et al. (23), 2018	3	OR	Not-short vs. Short	1.20 (0.93–1.55)	1.13 (1.01–1.28)
Coronary artery disease	Yang et al. (24), 2015	10	RR	Short vs. Ref	1.10 (1.04–1.17)	1.07 (1.03–1.12)
		10	RR	Long vs. Ref	1.03 (0.91–1.16)	1.00 (0.93–1.08)
Coronary heart disease	Yin et al. (25), 2017	14	RR	Per 1-h reduction	1.07 (1.03–1.12)	1.05 (1.03–1.08)
		12	RR	Per 1-h increment	1.05 (1.00–1.10)	1.07 (1.04–1.09)
Hypertension	Meng et al. (26), 2013	5	RR	Long vs. Ref	0.96 (0.76–1.21)	1.03 (0.92–1.15)
	Li et al. (27), 2019	7	RR	≤ 5 vs. 7 h	1.33 (1.04–1.70)	1.20 (1.13–1.26)
		3	RR	6 vs. 7 h	1.09 (1.04–1.14)	1.09 (1.04–1.14)
		6	RR	9 vs. 7 h	0.93 (0.85–1.01)	0.94 (0.90–0.97)
		4	RR	> 9 vs. 7 h	0.96 (0.75–1.24)	0.89 (0.80.0.99)
Total cardiovascular disease	Yin et al. (25), 2017	16	RR	Per 1-h reduction	1.06 (1.03–1.09)	1.04 (1.03–1.06)
		17	RR	Per 1-h increment	1.12 (1.08–1.16)	1.10 (1.09–1.12)
	Kwok et al. (28), 2018	5	RR	Short vs. Ref	1.16 (0.95–1.40)	1.08 (0.99–1.17)
**Diseases of the nervous system**						
Dementia	Fan et al. (29), 2019	7	HR	Long vs. Normal	1.77 (1.32–2.37)	1.55 (1.35–1.77)
		7	HR	Short vs. Normal	1.20 (0.91–1.59)	1.01 (0.94–1.09)
Alzheimer's disease	Fan et al. (29), 2019	6	HR	Long vs. Normal	1.63 (1.24–2.13)	1.59 (1.36–1.85)
		6	HR	Short vs. Normal	1.18 (0.91–1.54)	1.02 (0.94–1.10)
Cognitive decline	Wu et al. (30), 2017	3	RR	Shortest vs. Ref	1.37 (1.18–1.60)	1.37 (1.18–1.60)
		3	RR	Longest vs. Ref	1.17 (0.97–1.41)	1.17 (0.97–1.41)
Cognitive disorders	Wu et al. (30), 2017	9	RR	Shortest vs. Ref	1.33 (1.16–1.54)	1.32 (1.19–1.47)
		9	RR	Longest vs. Ref	1.20 (1.06–1.36)	1.20 (1.06–1.36)
Mild cognitive impairment/Dementia	Wu et al. (30), 2017	6	RR	Shortest vs. Ref	1.27 (0.97–1.66)	1.23 (1.05–1.44)
Mild cognitive impairment/Dementia	Wu et al. (30), 2017	6	RR	Longest vs. Ref	1.19 (1.00–1.42)	1.19 (1.01–1.41)
	Liang et al. (31), 2018	4	RR	Per 1-h increment	0.98 (0.97–1.00)	0.98 (0.97. 1.00)
Stroke	Leng et al. (32), 2015	12	RR	Short vs. Average	1.15 (1.07–1.24)	1.15 (1.07–1.24)
		12	RR	Long vs. Average	1.44 (1.26–1.65)	1.41 (1.32–1.52)
	He et al. (33), 2016	12	RR	Every 1-h reduction	1.10 (0.98–1.23)	1.05 (0.98–1.14)
		12	RR	Every 1-h increment	1.38 (1.23–1.55)	1.34 (1.25–1.44)
**Endocrine diseases**						
Diabetes	Holliday et al. (34), 2013	10	OR	Short vs. Ref	1.35 (1.19–1.53)	1.33 (1.20–1.48)
Type 2 diabetes	Cappuccio et al. (35), 2010	7	RR	Short vs. Ref	1.29 (1.03–1.60)	1.14 (1.02–1.27)
		6	RR	Long vs. Ref	1.48 (1.12–1.96)	1.38 (1.17–1.63)
	Shan et al. (36), 2015	9	RR	Per 1-h reduction	1.09 (1.04–1.15)	1.06 (1.04–1.08)
**Mortality**						
All cancer mortality	Li et al. (37), 2019	14	RR	Short vs. Ref	1.02 (0.99–1.05)	1.02 (0.99–1.05)
		14	RR	Long vs. Ref	1.05 (1.02–1.08)	1.05 (1.02–1.08)
	Stone et al. (38), 2019	18	HR	Lowest vs. Ref	1.04 (1.00–1.08)	1.03 (1.00–1.06)
		20	HR	Longest vs. Ref	1.09 (1.04–1.15)	1.08 (1.04–1.13)
All-cause mortality	Yin et al. (25), 2017	24	RR	Per 1-h reduction	1.06 (1.04–1.07)	1.04 (1.03–1.04)
		27	RR	Per 1-h increment	1.13 (1.11–1.15)	1.12 (1.11–1.13)
	Francesco et al. (39), 2010	16	RR	Long vs. Ref	1.29 (1.22–1.38)	1.24 (1.21–1.26)
Cardiovascular mortality	Kwok et al. (28), 2018	3	RR	Short vs. Ref	1.18 (0.90–1.53)	1.18 (0.90–1.53)
Mortality of coronary artery disease	Yang et al. (24), 2015	5	RR	Short vs. Ref	1.25 (1.06–1.47)	1.25 (1.12–1.41)
		5	RR	Long vs. Ref	1.26 (1.11–1.42)	1.24 (1.13–1.35)
Mortality of coronary heart disease	Kwok et al. (28), 2018	3	RR	Short vs. Ref	1.44 (0.74–2.83)	1.51 (1.05–2.16)
	Kwok et al. (28), 2018	5	RR	Short vs. Ref	1.29 (1.10–1.51)	1.29 (1.17–1.43)
	Kwok et al. (28), 2018	4	RR	Short vs. Ref	1.12 (0.98–1.27)	1.12 (1.01–1.24)
	Kwok et al. (28), 2018	6	RR	Long vs. Ref	1.36 (1.17–1.59)	1.39 (1.26–1.53)
Mortality of coronary heart disease	Kwok et al. (28), 2018	3	RR	Long vs. Ref	1.24 (1.00–1.53)	1.24 (1.00–1.53)
Mortality (all-cause and cause-specific)	Gallicchio et al. (40), 2009	16	RR	Short vs. Ref	1.10 (1.06–1.15)	1.10 (1.08–1.12)
Prostate cancer mortality	Liu et al. (41), 2020	6	RR	Short vs. Ref	0.99 (0.91–1.07)	0.99 (0.91–1.07)
		6	RR	Long vs. Ref	0.88 (0.75–1.04)	0.94 (0.87–1.02)
Stroke mortality	Li et al. (42), 2016	4	RR	Per 1-h reduction	1.05 (0.99–1.11)	1.05 (0.99–1.11)
		11	RR	Per 1-h increment	1.17 (1.13–1.20)	1.17 (1.13–1.20)
**Neoplasms**						
Breast cancer	Wong et al. (43), 2020	15	RR	Short vs. Ref	0.99 (0.97–1.01)	0.99 (0.98–1.01)
		15	RR	Long vs. Ref	1.00 (0.96–1.04)	1.01 (0.98–1.04)
Cancer	Lu et al. (44), 2013	9	RR	Short vs. Ref	1.05 (0.90–1.24)	1.02 (0.93–1.11)
		9	RR	Long vs. Ref	0.92 (0.76–1.12)	0.97 (0.88–1.06)
	Zhao et al. (45), 2013	9	HR	Per 1-h reduction	1.06 (0.92–1.22)	1.01 (0.94–1.09)
		12	HR	Per 1-h increment	0.91 (0.78–1.07)	0.98 (0.91–1.06)
**Nutritional diseases**						
Obesity	Miller et al. (46), 2020	13	RR	Per an additional hour	1.54 (1.33–1.77)	1.51 (1.43–1.60)
	Wu et al. (47), 2015	13	OR	Short vs. Ref	1.71 (1.36–2.14)	2.18 (2.13–2.22)
Overweight	Ruan et al. (48), 2015	7	OR	Lowest vs. Highest	1.79 (1.39–2.31)	2.22 (2.17–2.26)
Overweight and obesity	Fatima et al. (49), 2015	11	OR	Short vs. Ref	1.56 (1.24–1.98)	2.20 (2.16–2.25)
Overweight or obesity	Miller et al. (50), 2018	7	RR	Highest vs. Lowest	1.40 (1.18–1.65)	1.28 (1.16–1.41)
		8	RR	Highest vs. Lowest	1.57 (1.40–1.76)	1.61 (1.51–1.72)
**Other outcomes**						
Depression	Zhai et al. (51), 2015	6	RR	Short vs. Ref	1.30 (1.04–1.64)	1.30 (1.04–1.64)
		4	RR	Long vs. Ref	1.41 (1.04–1.92)	1.41 (1.04–1.92)
Dyslipidaemia	Kruisbrink et al. (52), 2017	6	RR	Short vs. Ref	1.01 (0.92–1.11)	1.05 (0.99–1.10)
		6	RR	Long vs. Ref	0.98 (0.87–1.10)	0.94 (0.88–1.00)
Gestational diabetes mellitus	Zhang et al. (53), 2020	4	RR	Long vs. Normal	1.19 (1.05–1.35)	1.19 (1.05–1.35)
Gestational diabetes mellitus	Zhang et al. (53), 2020	4	RR	Short vs. Not-short	2.02 (1.31–3.11)	2.02 (1.31–3.11)
	Xu et al. (54), 2018	5	OR	Short vs. Ref	1.58 (0.99–2.52)	1.37 (1.05–1.80)
		3	OR	Long vs. Ref	1.28 (1.10–1.49)	1.28 (1.10–1.49)
The developing or dying of coronary heart disease	Cappuccio et al. (55), 2011	7	RR	Short vs. Ref	1.48 (1.22–1.80)	1.48 (1.31–1.68)
		7	RR	Long vs. Ref	1.38 (1.15–1.66)	1.41 (1.26–1.59)
The developing or dying of stroke	Cappuccio et al. (55), 2011	4	RR	Short vs. Ref	1.15 (1.00–1.32)	1.15 (1.00–1.32)
		4	RR	Long vs. Ref	1.65 (1.45–1.87)	1.65 (1.45–1.87)
The developing or dying of total cardiovascular disease	Cappuccio et al. (55), 2011	7	RR	Short vs. Ref	1.03 (0.93–1.15)	1.03 (0.93–1.15)
		8	RR	Long vs. Ref	1.41 (1.20–1.67)	1.42 (1.30–1.54)
**Sleep quality**						
All-cause mortality	Kwok et al. (28), 2018	10	RR	Poor vs. Good	1.03 (0.93–1.14)	1.03 (0.98–1.09)
Cardiovascular mortality	Kwok et al. (28), 2018	4	RR	Poor vs. Good	0.96 (0.82–1.13)	0.96 (0.82–1.13)
Coronary heart disease	Kwok et al. (28), 2018	4	RR	Poor vs. Good	1.44 (1.09–1.90)	1.32 (1.12–1.56)
Diabetes mellitus	Anothaisintawee et al. (56), 2015	11	RR	Poor vs. Not- Poor	1.40 (1.21–1.63)	1.32 (1.28–1.36)
Gestational diabetes mellitus	Zhang et al. (53), 2020	4	RR	Poor vs. Not- Poor	1.27 (1.11–1.44)	1.27 (1.11–1.44)
Inflammatory bowel disease	Hao et al. (57), 2020	3	OR	Poor vs. Not- Poor	2.54 (1.37–4.71)	2.38 (1.71–3.31)
Preterm birth	Wang et al. (58), 2020	5	RR	Poor vs. Good	1.54 (1.18–2.00)	1.26 (1.15–1.38)

*CI, confidence interval; OR, odds ratio; RR, relative risk; HR: hazard ratio*.

*All statistical tests were two-sided*.

### Summary Effect Size

Of the 78 meta-analyses from 32 articles regarding sleep duration, the summary random effects estimates were significant at *P* < 1 × 10^−6^ in 10 (13%) meta-analyses, and the summary fixed effects estimates were significant in 28 (36%) meta-analyses (Supplementary Table S4). Thirty-nine (50%) meta-analyses reported that the largest study effect was nominally statistically significant, with a *P* < 0.05, and a more conservative effect than the summary random effects was observed in 57 (73%) meta-analyses. The studies with the smallest SE for each association suggested that 30 of 78 were significant at *P* < 0.05.

Out of the 7 meta-analyses from 5 articles regarding sleep quality, the summary fixed-effects and random-effects estimates were significant at *P* < 0.05. However, when we used (*P* < 1 × 10^−6^) as a threshold for significance, the summary random effects estimates were not significant in any meta-analyses, and 3 (43%) meta-analyses produced significant summary results using the fixed-effects methods (Supplementary Table S4). The studies with the smallest standard error for each association suggested that 5 of 7 were significant at *P* < 0.05, and a more conservative effect than the summary random effects was observed in 5 (71%) meta-analyses. The studies with the smallest SE for each association suggested that two of 7 were significant at *P* < 0.05.

### Heterogeneity and Prediction Intervals

In all the included studies regarding sleep duration, approximately 44 (56%) studies had a lower heterogeneity, with I^2^ ≤ 50%; approximately 28 (36%) studies had substantial heterogeneity estimates, with I^2^ between 50 and 75%; and 6 (8%) studies had considerable heterogeneity estimates, with I^2^ > 75% (Table 2). When 95% PIs were evaluated, we found that 10 meta-analyses [cognitive disorders, stroke, diabetes, all cancer mortality, all-cause mortality, mortality (both all-cause and cause-specific), stroke mortality, obesity, overweight and/or obesity, and the development or death of stroke] excluded the null value (Table 2).

**Table 2:** Level of evidence for the association of sleep duration/quality for health outcomes.

**Outcomes**	**Level of comparison**	**Features used for classification of level of evidence**								
		**Significance threshold reached** ^*^	**I^**2**^ (95% CI)**	**95% prediction interval**	**Egger's *P* value**	**Excess significance** ^**§**^		**Largest study Significant**	**Small-study effect/Excess significant bias**	**Evidence class**
						**O/E** ^**#**^	***P*** **value**^**¶**^			
**Sleep duration**										
**Diseases of the circulatory system**										
Atrial fibrillation	Not-short vs. Short	>0.05	66.1 (0–90)	(0.07–20.51)	0.607	1/0.86	0.860	No	No/No	No association
Coronary artery disease	Short vs. Ref	<0.001 but >10^−6^	24.9 (0–62)	(0.98–1.25)	0.019	4/3.01	0.495	No	Yes/No	Suggestive
	Long vs. Ref	>0.05	45.8 (0–72)	(0.75–1.42)	0.477	3/4.22	0.435	Yes	No/No	No association
Coronary heart disease	Per 1-h reduction	<0.05 but >0.001	58 (29–75)	(0.93–1.23)	0.097	5/5.09	0.961	No	Yes/No	Weak
	Per 1-h increment	<0.05 but >0.001	64.5 (39–79)	(0.89–1.23)	0.186	6/4.20	0.275	Yes	No/No	Weak
Hypertension	Long vs. Ref	>0.05	48.2 (0–79)	(0.52–1.76)	0.147	1/1.42	0.677	No	No/No	No association
	≤ 5 vs. 7 h	<0.05 but >0.001	79.2 (57–90)	(0.63–2.84)	0.450	4/2.93	0.413	Yes	No/No	Weak
	6 vs. 7 h	<0.001 but >10^−6^	0 (0–90)	(0.82–1.45)	0.872	1/0.17	0.041	Yes	No/Yes	Suggestive
	9 vs. 7 h	>0.05	6.5 (0–76)	(0.78–1.10)	0.445	2/0.93	0.228	Yes	No/No	No association
	> 9 vs. 7 h	>0.05	63 (0–88)	(0.36–2.60)	0.547	1/1.04	0.960	Yes	No/No	No association
Total cardiovascular disease	Per 1-h reduction	<0.001 but >10^−6^	51.4 (20–71)	(0.98–1.14)	0.050	8/4.98	0.103	Yes	Yes/No	Suggestive
	Per 1-h increment	<10^−6^	75.5 (63–84)	(0.97–1.29)	0.239	12/12.70	0.696	No	No/No	Weak
	Short vs. Ref	>0.05	54.6 (0–82)	(0.70–1.91)	0.100	2/1.55	0.648	No	Yes/No	No association
**Diseases of the nervous system**										
Dementia	Long vs. Normal	<0.001 but >10^−6^	68.3 (30–86)	(0.74–4.20)	0.206	5/2.32	0.032	Yes	No/Yes	Suggestive
	Short vs. Normal	>0.05	62.2 (14–83)	(0.54–2.66)	0.212	2/2.02	0.990	No	No/No	No association
Alzheimer's disease	Long vs. Normal	<0.001 but >10^−6^	45.1 (0–78)	(0.79–3.33)	0.761	4/1.37	0.011	Yes	No/Yes	Suggestive
	Short vs. Normal	>0.05	57.8 (0–83)	(0.57–2.47)	0.148	0/1.78	0.112	No	No/No	No association
Cognitive decline	Shortest vs. Ref	<0.001 but >10^−6^	0 (0–90)	(0.51–3.71)	0.652	2/0.34	0.003	Yes	No/Yes	Suggestive
	Longest vs. Ref	>0.05	0 (0–90)	(0.35–3.92)	0.714	0/0.18	0.662	No	No/No	No association
Cognitive disorders	Shortest vs. Ref	<0.001 but >10^−6^	25.6 (0–63)	(0.98–1.81)	0.304	4/1.89	0.084	Yes	No/Yes	Suggestive
	Longest vs. Ref	<0.05 but >0.001	0 (0–60)	(1.04–1.39)	0.048	1/1.59	0.608	No	Yes/No	Weak
Mild cognitive impairment/ Dementia	Shortest vs. Ref	>0.05	46.3 (0–79)	(0.64–2.53)	0.605	2/1.28	0.476	Yes	No/No	No association
	Longest vs. Ref	<0.05 but >0.001	2.7 (0–75)	(0.91–1.56)	0.241	0/0.96	0.284	No	No/No	Weak
	Per 1-h increase	>0.05	0 (0–85)	(0.95–1.02)	0.872	1/0.87	0.879	No	No/No	No association
Stroke	Short vs. Average	<0.001 but >10^−6^	0 (0–50)	(1.06–1.25)	0.082	2/2.43	0.759	No	Yes/No	Suggestive
	Long vs. Average	<10^−6^	68 (42–82)	(0.93–2.23)	0.486	9/2.73	0.000	Yes	No/Yes	Highly suggestive
	Every 1-h decrease	>0.05	44.2 (0–70)	(0.80–1.52)	0.080	2/4.56	0.128	No	Yes/No	No association
	Every 1-h increase	<10^−6^	56.3 (22–75)	(0.95–2.00)	0.089	10/11.55	0.018	No	Yes/No	Weak
**Endocrine diseases**										
Diabetes	Short vs. Ref	<0.001 but >10^−6^	16.6 (0–58)	(1.06–1.71)	0.021	5/1.84	0.010	Yes	Yes/Yes	Suggestive
Type 2 diabetes	Short vs. Ref	<0.05 but >0.001	57.5 (11–80)	(0.72–2.29)	0.141	3/3.47	0.722	No	No/No	Weak
	Long vs. Ref	<0.05 but >0.001	37.9 (0–74)	(0.77–2.84)	0.421	3/1.56	0.180	Yes	No/No	Weak
	Per 1-h reduction	<0.001 but >10^−6^	61.8 (21–82)	(0.97–1.24)	0.083	7/2.43	0.001	Yes	Yes/Yes	Suggestive
**Mortality**										
All cancer mortality	Short vs. Ref	>0.05	0 (0–52)	(0.99–1.05)	0.819	0/2.08	0.118	No	No/No	No association
	Long vs. Ref	<0.001 but >10^−6^	0 (0–52)	(1.02–1.08)	0.001	2/3.01	0.511	No	Yes/No	Suggestive
All cancer mortality	Lowest vs. Ref	>0.05	19.5 (0–54)	(0.95–1.14)	0.136	2/5.41	0.080	No	No/No	No association
	Longest vs. Ref	<0.001 but >10^−6^	18.8 (0–53)	(0.98–1.21)	0.096	4/3.87	0.942	Yes	Yes/No	Suggestive
All-cause mortality	Per 1-h reduction	<10^−6^	57.4 (36–71)	(1.01–1.11)	0.001	15/9.02	0.012	Yes	Yes/Yes	Highly suggestive
	Per 1-h increment	<10^−6^	77.4 (69–83)	(1.05–1.22)	0.423	27/12.36	1.557 ×10^8^	Yes	No/Yes	Highly suggestive
	Long vs. Ref	<10^−6^	71 (57–80)	(1.03–1.62)	0.160	15/8.96	0.002	Yes	No/Yes	Highly suggestive
Cardiovascular mortality	Short vs. Ref	>0.05	0 (0–90)	(0.21–6.44)	0.028	0/0.25	1.000	No	Yes/No	No association
Mortality of coronary artery disease	Short vs. Ref	<0.05 but >0.001	42.6 (0–75)	(0.82–1.90)	0.644	4/1.88	0.050	Yes	No/Yes	Weak
	Long vs. Ref	<0.001 but >10^−6^	38.9 (0–73)	(0.93–1.70)	0.493	3/3.13	0.901	No	No/No	Suggestive
Mortality of coronary heart disease	Short vs. Ref	>0.05	55.5 (0–87)	(0.00–1,535.63)	0.667	1/0.73	0.569	No	No/No	No association
	Short vs. Ref	<0.05 but >0.001	55.2 (0–81)	(0.83–2.01)	0.907	3/2.40	0.676	Yes	No/No	Weak
	Short vs. Ref	>0.05	24.3 (0–68)	(0.84–1.49)	0.851	1/1.10	1.000	No	No/No	No association
	Long vs. Ref	<0.001 but >10^−6^	53.3 (1.0–78)	(0.89–2.09)	0.706	5/3.99	0.671	Yes	No/No	Suggestive
	Long vs. Ref	>0.05	0 (0–90)	(0.31–4.97)	0.291	1/0.50	0.419	Yes	No/No	No association
Mortality (all-cause and cause-specific)	Short vs. Ref	<0.001 but >10^−6^	20.5 (0–56)	(1.01–1.21)	0.712	4/2.95	0.500	Yes	No/No	Suggestive
Prostate cancer mortality	Short vs. Ref	>0.05	0 (0–75)	(0.88–1.10)	0.466	0/0.86	0.316	No	No/No	No association
	Long vs. Ref	>0.05	55.6 (0–82)	(0.56–1.40)	0.241	2/1.52	0.651	No	No/No	No association
Stroke mortality	Per 1-h reduction	>0.05	0 (0–71)	(0.98–1.12)	0.996	1/1.27	0.774	No	No/No	No association
	Per 1-h increase	<10^−6^	1.5 (0–61)	(1.12–1.21)	0.490	8/1.92	1.001 × 10^7^	Yes	No/Yes	Highly suggestive
**Neoplasms**										
Breast cancer	Short vs. Ref	>0.05	7.5 (0–44)	(0.96–1.03)	0.347	2/2.57	0.695	No	No/No	No association
	Long vs. Ref	>0.05	11.2 (0–49)	(0.93–1.07)	0.065	0/2.69	0.070	No	Yes/No	No association
Cancer	Short vs. Ref	>0.05	57.6 (14–79)	(0.67–1.66)	0.349	3/3.10	0.944	No	No/No	No association
	Long vs. Ref	>0.05	68.9 (40–84)	(0.52–1.66)	0.374	4/3.51	0.740	No	No/No	No association
	Per 1-h reduction	>0.05	63.8 (28–82)	(0.69–1.62)	0.374	3/3.33	0.820	No	No/No	No association
	Per 1-h increase	>0.05	67.6 (42–82)	(0.55–1.52)	0.071	5/4.80	0.908	No	Yes/No	No association
**Nutritional diseases**										
Obesity	Per an additional hour	<10^−6^	68.6 (44–82)	(1.00–2.37)	0.710	9/3.53	0.001	Yes	No/Yes	Highly suggestive
	Short vs. Ref	<0.001 but >10^−6^	91.3 (87–94)	(0.73–4.00)	0.067	10/5.62	0.014	Yes	Yes/Yes	Suggestive
Overweight	Lowest vs. Highest	<0.001 but >10^−6^	76.5 (53–88)	(0.85–3.78)	0.167	7/3.09	0.003	Yes	No/Yes	Suggestive
Overweight and obesity	Short vs. Ref	<0.001 but >10^−6^	87.1 (80–92)	(0.69–3.56)	0.026	10/5.75	0.010	Yes	Yes/Yes	Suggestive
Overweight or obesity	Highest vs. Lowest	<0.001 but >10^−6^	40.5 (0–75)	(0.94–2.09)	0.002	5/2.00	0.012	Yes	Yes/Yes	Suggestive
	Highest vs. Lowest	<10^−6^	22.7 (0–64)	(1.23–2.00)	0.521	6/1.49	4.063 × 10^5^	Yes	No/Yes	Highly suggestive
**Other outcome**										
Depression	Short vs. Ref	<0.05 but >0.001	0 (0–71)	(0.97–1.76)	0.965	1/1.09	0.925	No	No/No	Weak
	Long vs. Ref	<0.05 but >0.001	0 (0–79)	(0.86–2.32)	0.526	0/0.77	0.328	No	No/No	Weak
Dyslipidaemia	Short vs. Ref	>0.05	62.2 (29–80)	(0.75–1.36)	0.147	4/4.57	0.585	Yes	No/No	No association
	Long vs. Ref	>0.05	63.9 (38–79)	(0.65–1.48)	0.249	3/5.79	6.291 × 10^10^	No	No/No	No association
Gestational diabetes mellitus	Long vs. Normal	<0.05 but >0.001	0 (0–85)	(0.90–1.58)	0.883	1/0.67	0.657	Yes	No/No	Weak
	Short vs. Not-short	<0.001 but >10^−6^	0 (0–85)	(0.78–5.19)	0.003	1/0.58	0.545	No	Yes/No	Weak
	Short vs. Ref	>0.05	57.1 (0–83)	(0.42–5.93)	0.031	3/1.90	0.312	No	Yes/No	No association
	Long vs. Ref	<0.001 but >10^−6^	0 (0–85)	(0.92–1.78)	0.771	1/0.36	0.260	Yes	No/No	Suggestive
The developing or dying of coronary heart disease	Short vs. Ref	<0.001 but >10^−6^	43.3 (0–72)	(0.91–2.41)	0.962	6/3.23	0.036	Yes	No/Yes	Suggestive
	Long vs. Ref	<0.001 but >10^−6^	49 (1–74)	(0.84–2.29)	0.913	5/4.96	0.976	Yes	No/No	Suggestive
The developing or dying of stroke	Short vs. Ref	<0.05 but >0.001	0 (0–75)	(0.95–1.39)	0.304	0/0.82	0.310	No	No/No	Weak
	Long vs. Ref	<10^−6^	0 (0–75)	(1.38–1.97)	0.955	4/0.47	4.725 × 10^8^	Yes	No/Yes	Highly suggestive
The developing or dying of total cardiovascular disease	Short vs. Ref	>0.05	0 (0–60)	(0.92–1.16)	0.470	0/1.01	0.279	No	No/No	No association
	Long vs. Ref	<0.001 but >10^−6^	59.8 (26–78)	(0.86–2.31)	0.794	6/2.89	0.022	Yes	No/Yes	Suggestive
**Sleep quality**										
All-cause mortality	Poor vs. Good	>0.05	0.6 (14–79)	(0.79–1.35)	0.917	3/2.89	1.000	Yes	No/No	No association
Cardiovascular mortality	Poor vs. Good	>0.05	0 (0–85)	(0.67–1.37)	0.303	0/0.31	1.000	No	No/No	No association
Coronary heart disease	Poor vs. Good	<0.05 but >0.001	53.2 (0–85)	(0.51–4.10)	0.075	1/1.08	1.000	No	Yes/No	Weak
Diabetes mellitus	Poor vs. Not- Poor	<0.001 but >10^−6^	84.1 (73–91)	(0.89–2.22)	0.679	8/4.04	0.013	Yes	No/Yes	Suggestive
Gestational diabetes mellitus	Poor vs. Not- Poor	<0.001 but >10^−6^	0 (0–85)	(0.95–1.68)	0.807	2/0.50	0.024	Yes	No/Yes	Suggestive
Inflammatory bowel disease	Poor vs. Not- Poor	<0.05 but >0.001	62.3 (0–89)	(0–1,940.14)	0.683	2/0.87	0.148	Yes	No/No	Weak
Preterm birth	Poor vs. Good	<0.001 but >10^−6^	76.7 (43–90)	(0.67–3.51)	0.039	5/1.73	0.002	Yes	Yes/Yes	Weak

*CI, confidence interval*.

§ *Expected number of statistically significant studies using the point estimate of the largest study (smallest standard error) as the plausible effect size*.

^*^ *P value under the random-effects model*.

# *Observed/Expected number of statistically significant studies*.

¶ *P value of the excess statistical significance test*.

*All statistical tests two sided*.

Of the 7 meta-analyses about sleep quality, 3 (42%) showed low heterogeneity (I^2^ < 50%), and 2 (29%) separately showed substantial heterogeneity (I^2^ > 50% and I^2^ ≤ 75%) and considerable heterogeneity (I^2^ > 75%; Table 2). When 95% PIs were evaluated, we found that no meta-analysis excluded the null value (Table 2).

### Small-Study Effects and Excess Significance Bias

According to Egger's test, evidence of small-study effects was observed in 21 (27%) of 78 meta-analyses and 2 (29%) of 7 meta-analyses about duration and quality of sleep, respectively (Table 2). When taking the largest study estimate as to the plausible effect size, 22 (28%) of 78 meta-analyses and 3 (43%) of 7 meta-analyses about duration and quality of sleep respectively showed evidence of excess significance (Table 2).

### Methodological Quality of the Meta-Analyses

**Figure 2:**
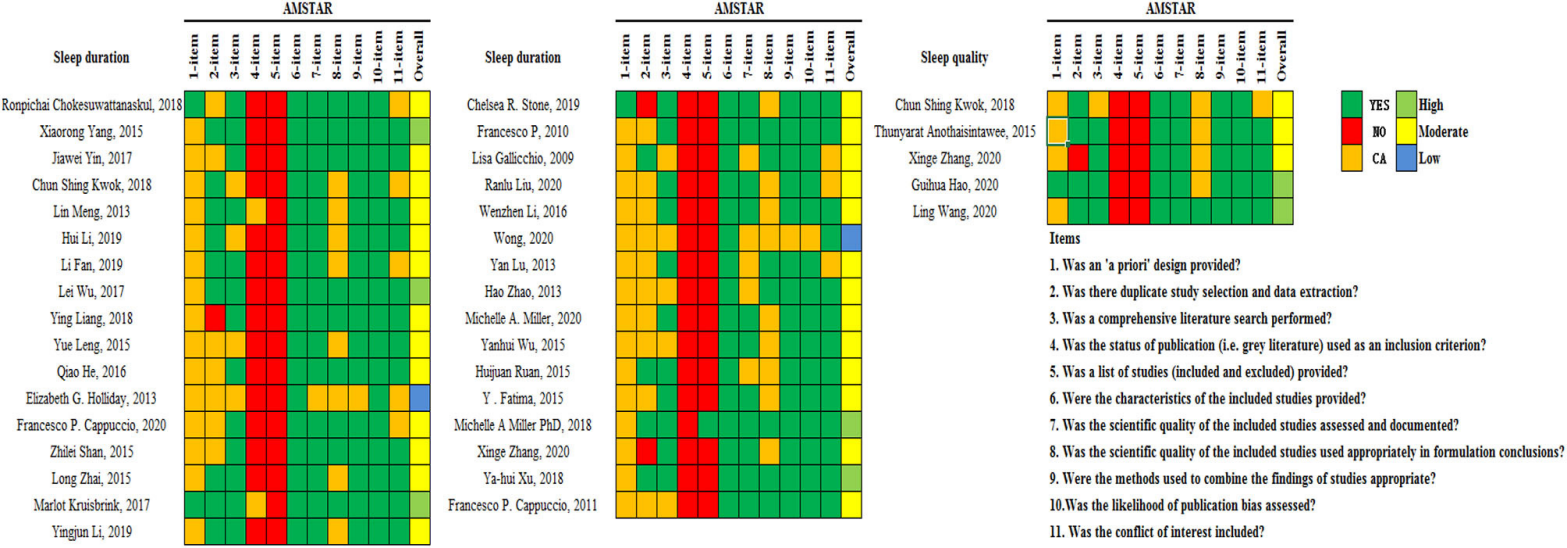
Detailed evaluation of the methodological quality with AMSTAR-1.

The methodological quality of the included studies regarding sleep duration (n = 33) and sleep quality (n = 5) was assessed by AMSTAR-1, which contained 11 items for scoring. Figure 2 provides a breakdown of AMSTAR-1 levels for studies representing each study. For sleep duration, the median AMSTAR-1 score achieved across all studies was 6 out of 11 (range from 2 to 9). The studies were rated at three levels: 15% were rated as “high,” 79% were rated as “moderate,” and 6% were classified as “low.” For sleep quality, the median AMSTAR-1 score achieved across all studies was 7 (range from 5 to 8). Approximately 40% were rated as being of “high,” 60% as “moderate” quality, and no meta-analysis was categorized into low quality according to the AMSTAR-1 criteria. The common flaws were that gray literature was not considered in the literature search (item 4), and the list of excluded studies was not presented (item 5).

### Evidence Grading

For sleep duration, no association presented convincing evidence, the only evidence of long sleep duration with an increased risk of all-cause mortality was categorized as highly suggestive, and the methodological quality was moderate (Table 2). Moreover, suggestive evidence supported the associations between long sleep and increased risk of 5 health outcomes (stroke, dyslipidaemia, mortality of coronary heart disease, stroke mortality, and the development or death of stroke); short sleep duration and increased risk of overweight and/or obesity. Moreover, 14 associations were supported by weak evidence. The remaining 31 associations were not confirmed. The detailed results of the analyses on which the evidence ratings were based are shown in Table 2.

For sleep quality, no association presented convincing or highly suggestive evidence, whereas suggestive evidence suggested that poor sleep quality was associated with an increased risk of diabetes mellitus and gestational diabetes mellitus. Moreover, 3 associations were supported by weak evidence and 2 associations were not confirmed.

## Discussion

In this umbrella review, to objectively assess the strength of associations between duration and quality of sleep and health outcomes, we performed a comprehensive overview by incorporating evidence from the current systematic reviews and meta-analyses of prospective studies. Overall, 85 published meta-analyses were included, and 52 (61%) were nominally statistically significant at *P* < 0.05 under the random-effects models. Although the study confirmed that short/long sleep duration or poor sleep quality was associated with an increase in the important health outcomes, the mechanisms do not seem straightforward.

In this umbrella review, evidence of the association of long sleep duration with an increased risk of all-cause mortality (25), was the only one categorized as highly suggestive, and the methodological quality was moderate in the above outcome. An association between long sleep and an increased risk of all-cause mortality was reported previously in studies with high quality and large sample sizes (59–62), which was consistent with our results. Heslop and colleagues (63), however, analyzed data from a workplace-based study of Scottish men and women who were followed over a 25-year period and found that long sleep was associated with decreased risk of all-cause mortality in men. However, this study reported RRs with only 3 quantitative categories of sleep duration. Meanwhile, long sleep duration was defined as >8 h, which may result in inaccurate evaluation of extremely long sleep. To date, no published studies have demonstrated a possible mechanism mediating the effect of long sleep as a cause of mortality. The association between a long duration of sleep and mortality may be explained by residual confounding and comorbidities (64). In particular, depressive symptoms, low socioeconomic status, low level of physical activity, unemployment, undiagnosed health conditions, poor general health, and cancer-related fatigue have all been shown to be associated with long sleep (64).

Suggestive evidence has shown that long sleep duration is positively linked with the morbidity of stroke (32) and mortality of stroke per 1-h increase in sleep duration (42). At present, the biological mechanisms of the relationship between long sleep and stroke are not clear. One important biological pathway is inflammation, as long sleep periods have been associated with an increased level of inflammatory biomarkers, such as C-reactive protein and interleukin-6 (65–68). Interestingly, a number of studies have associated long sleep with cardiovascular conditions including atrial fibrillation, carotid artery atherosclerosis, and left ventricular mass, which might have predisposed one to the risk of stroke (69–73). Meanwhile, some studies suggested an association for long sleep and stroke only among those with limited physical function (74) or with a history of hypertension (75). Another possible biological pathway is due to sleep disorders such as sleep-disordered breathing (76). Decreased cerebral blood flow and raised intracranial pressure occurred during apneic events in some studies (77, 78), and cerebral hypoperfusion may also occur during wakefulness in sleep apnea patients (79). Klingelhofer et al. (80) found that blood flow in the middle cerebral artery during apneic showed rapid increases and decreases in velocity. Such changes could incline vulnerable individuals to ischemic or hemorrhagic events (76). Several epidemiological studies have explored this association. A previous meta-analysis indicated that long sleep duration was associated with an increased risk of stroke (55), but they did not use a dose–response analysis to determine the association (55). A meta-analysis by Ge showed a significantly increased risk of stroke incidence and mortality at long sleep durations in both cohort and cross-sectional studies. Their subgroup analysis also showed that long sleep duration was a statistical stroke risk in both sexes and in Asians (81). Those results were in accordance with ours. However, the relationship between sleep duration and stroke may be related to stroke types (82), age (83), gender and race (84), and high-quality studies are therefore needed to explore this matter.

We also found suggestive evidence that long sleep duration is associated with an increased risk of mortality of coronary heart disease (28). Khan et al. (85) found that there is a significant association of coronary heart disease in the top quartile of sleep duration compared to those in the bottom quartile. Those results were in accordance with ours. However, further adjustment for risk factors including systolic blood pressure, history of cardiovascular disease, diabetes, smoking, alcohol use, renal function and serum Low-Density Lipoprotein cholesterol attenuated the associations with fatal coronary heart disease. However, the average sleep duration in Khan's study was 9.1 h with the lowest quartile being 8.2 h. This is longer than previously reported data from Western populations. Long sleep duration has been related to systemic inflammation, an increase in cytokines and changes in several metabolic pathways (68). The lack of physiological challenge due to increased sleep has also been proposed as a mechanism that may increase mortality (86). Longer sleep duration has also been linked to depression and other psychiatric disorders, which are known to be associated with increased cardiovascular disease events (51, 87). However, all these proposed mechanisms are speculative at best and require more research.

Suggestive evidence also showed that short sleep duration is linked with the increased risk of overweight or obesity only observed in children (50). There are several lines of evidence to suggest plausible mechanisms. Sleep deprivation is associated with various hormonal responses that may affect both hunger and satiety, leading to appetite dysregulation. These include lower leptin and higher ghrelin levels (88, 89), which would increase appetite. Sleep deprivation has effects on endocannabinoids which regulate a variety of central nervous system processes including appetite (90). Changes in factors that affect metabolism, including insulin and glucose metabolism, cortisol, growth hormone and thyroid stimulating hormone are also important (48, 91–95). In turn, obesity predisposes individuals to metabolic dysfunction that can cause sleep apnea, which leads to short sleep duration (96). Activation of inflammatory pathways by short sleep periods may be implicated in the development of obesity (97) and it can up and downregulate the expression of genes involved in oxidative stress and metabolism (98). Finally, insufficient sleep is associated with alterations in attention, impulse control, mood, motivation, and judgment, and all of these factors could potentially influence eating behaviors, energy intake, and ultimately BMI in children (99).

Regarding sleep quality, we found that poor sleep quality is associated with an increased risk of diabetes mellitus (56) and gestational diabetes mellitus (53). This result is consistent with the results of a previous meta-analysis, which concluded that sleep quality may be a novel and independent risk factor for poorer glycemic control in type 2 diabetes patients (100). Poor sleep quality as defined by the presence of one or more insomnia symptoms included in the Diagnostic and Statistical Manual of Mental Disorders diagnostic criteria was associated with a 40% increase in the risk of developing diabetes. Poor self-reported sleep quality may also be linked with other comorbid conditions, such as depression, undiagnosed obstructive sleep apnea and sleep deprivation, which are risk factors for diabetes. Adjusting for these covariates still resulted in a significant association between poor sleep quality and incident diabetes in several studies (101, 102). Pregnant women with poor sleep quality had an increased risk of gestational diabetes mellitus (GDM). In addition to its direct effect on GDM, sleep quality has been considered a moderator of the association between sleep duration and GDM risk (103). The potential pathophysiological mechanisms between poor sleep quality and glucose intolerance have been well established, including decreased brain glucose utilization (104–106), sympathetic nervous system overactivity (107, 108), alterations in the hypothalamic–pituitary–adrenal axis and growth hormone (109–111), elevated systemic inflammatory response (112, 113), reduction in the percentage of slow wave sleep (114), adipocyte dysfunction (115), changes in appetite-regulating hormones (88), and increased obesity risk (95).

To our knowledge, the present study is the first umbrella review to quantitatively evaluate the existing evidence of the associations between duration and quality of sleep and health outcomes. The main strength of our umbrella review was to provide a comprehensive summary and evaluation of the credibility and validity of evidence of duration and quality of sleep and health outcomes according to the assessment results of a series of statistical analyses. In addition, we searched three databases through a rigorous strategy, and two authors independently extracted the information. Moreover, we followed the AMSTAR-1 criteria to assess the methodological quality of selected studies in our umbrella review, and most of the investigated meta-analyses achieved a moderate-to-high quality score. We used standardized criteria to explore the extent of heterogeneity and potential bias among the included studies and further assessed the strength of claimed associations to identify which was the most credible evidence. We also used the criteria of evidence grading to evaluate the evidence categorization.

Nevertheless, several limitations should be noted when interpreting the results. Firstly, we failed to find convincing evidence for the relation of sleep and health outcomes. In addition, the quality of the evidence was rated weak or not confirmed for 39% of the associations (n = 33). Thus, further research is needed for outcomes for which the certainty of evidence was rated weak or not confirmed. Secondly, owing to the limited studies, we failed to conduct subgroup analysis (e.g., exploring by age, sex, geographical location), or sensitive analysis (e.g., excluding studies with high risks), and other relevant factors might have been missed. Many meta-analyses lacked dose-response information and compared high vs. low sleep duration without defining thresholds for these categories. Thirdly, we only evaluated published meta-analyses of prospective studies with available data, therefore, meta-analyses of randomized controlled trials were not included in our study. Fourthly, for sleep quality, only systematic reviews and meta-analyses assessing sleep quality by the PSQI questionnaire were included in this umbrella review. The PSQI is currently the only standardized clinical instrument that covers a broad range of indicators relevant to sleep quality (116). Lastly, we did not examine any error of the meta-analyses or the quality of the primary studies, as these were beyond the scope of our umbrella review. Our findings appear to be very convincing, but one may need to practice caution in terms of considering the implications of the results in the community. Although long or short sleep is associated with an increased risk of some health outcomes, there is no rigorous evidence that lengthening or shortening sleep duration can lead to a smaller frequency of these outcomes.

In conclusion, abnormal duration or quality of sleep was significantly associated with an extensive range of adverse health-related outcomes. Based on our umbrella review, although 36 studies explored 36 unique associations, the highly suggestive evidence only supported that long sleep duration was associated with an increased risk of all-cause mortality. The relationship between abnormal duration or quality of sleep and other outcomes could be genuine, but there is still limited evidence for them. Overall, this article assessed the associations between duration and quality of sleep and health outcomes based on previous studies, which is helpful for identifying at-risk groups and developing prevention strategies to counteract the effect of sleep discrepancies. Abnormal duration or quality of sleep is harmful to human health, but further high-quality prospective studies and better designed trials are needed to generate definite conclusions.

## Data Availability Statement

The original contributions presented in the study are included in the article/Supplementary Material, further inquiries can be directed to the corresponding authors.

## Author Contributions

T-TG and Q-JW contributed to the study design. CG and X-YL conducted the literature search. JG, J-LL, X-YL, F-HL, and MZ extracted the data and conducted the analyses. CG, JG, T-TG, J-LL, Y-TS, and Y-HZ wrote the first draft of the manuscript and edited the manuscript. All authors read and approved the final manuscript and accept responsibility for the integrity of the data analyzed.

## Funding

This work was supported by the National Key R&D Program of China (No. 2017YFC0907403 to Y-HZ), Natural Science Foundation of China (Nos. 82073647 and 81602918 to Q-JW and No. 82103914 to T-TG), LiaoNing Revitalization Talents Program (No. XLYC1907102 to Q-JW), Shenyang High-level Innovative Talents Support Program (No. RC190484 to Q-JW), and 345 Talent Project of Shengjing Hospital of China Medical University (Q-JW and T-TG).

## Conflict of Interest

The authors declare that the research was conducted in the absence of any commercial or financial relationships that could be construed as a potential conflict of interest.

## Publisher's Note

All claims expressed in this article are solely those of the authors and do not necessarily represent those of their affiliated organizations, or those of the publisher, the editors and the reviewers. Any product that may be evaluated in this article, or claim that may be made by its manufacturer, is not guaranteed or endorsed by the publisher.
